# The Association Between COVID-19 Information Sources and Stigma Against Health Care Workers Among College Students: Cross-sectional, Observational Study

**DOI:** 10.2196/35806

**Published:** 2022-07-07

**Authors:** Miharu Nakanishi, Mai Sakai, Gen Takagi, Keita Toshi, Koubun Wakashima, Hatsumi Yoshii

**Affiliations:** 1 Department of Psychiatric Nursing Tohoku University Graduate School of Medicine Sendai-shi Japan; 2 Research Center for Social Science & Medicine Tokyo Metropolitan Institute of Medical Science Setagaya-ku Japan; 3 Department of Welfare Psychology Faculty of General Welfare Tohoku Fukushi University Sendai-shi Japan; 4 Department of Clinical Psychology & Family Psychology Graduate School of Education Tohoku University Sendai-shi Japan

**Keywords:** health personnel, social media, social stigma, young adult, Twitter, public health, COVID-19, health care workers, students, information accuracy, information credibility, dissemination, information source, misinformation, information spread, infodemiology

## Abstract

**Background:**

The COVID-19 pandemic has triggered stigmatic attitudes against health care workers. Some forms of social media may play a role in disseminating stigmatizing messages.

**Objective:**

We aimed to investigate the association between COVID-19 information sources and stigma against health care workers among college students during the pandemic.

**Methods:**

A cross-sectional, observational study was conducted using a web-based platform in the Tohoku region of Japan. College students aged ≥20 years were asked to complete the questionnaire between August 18 and October 31, 2020. Stigma against health care workers was evaluated using a modified Japanese version of the Social Distance Scale. Participants were also asked to rate their perceived vulnerability to infection using the Japanese version of the Perceived Vulnerability to Disease scale.

**Results:**

A total of 281 students from 8 colleges completed the web-based survey. There were 139 (49.5%) participants who used Twitter, 187 (66.5%) who used news websites, and 46 (16.4%) who used the websites of public health agencies as COVID-19 information sources. After adjusting for age, sex, department, and Perceived Vulnerability to Disease scores, the level of stigma did not differ between students who used Twitter and those who did not. Students who used the websites of public health agencies showed a significantly less stigmatic attitude than those who did not.

**Conclusions:**

Fact-checking and directing visitors to credible information sources from public health agencies may have prevented the formation of stigmatic attitudes toward health care workers. An effective strategy to enable easy access to information provided by public agencies should be integrated into widespread web-based platforms.

## Introduction

The COVID-19 pandemic is a serious threat to public health. Moreover, it is characterized by widespread fear, worry, and uncertainty, as many COVID-19 infections are contracted through presymptomatic and asymptomatic transmissions [1]. This health emergency has triggered discriminatory behavior and stigma against health care workers, despite their vital role in caring for people with COVID-19 [2,3]. Stigma is defined as an undesirable characteristic that results in discrimination against an individual [4]. Several incidents of stigmatization of health care workers have been reported; these include avoidance by family members or their community, being denied access to public transport, and even being subjected to physical assault [3,5]. The psychological challenges entailing stigmatization may amplify the negative consequences, such as emotional burnout [6], posttraumatic stress disorder [7], and turnover [8] of working with COVID-19 patients as frontline care providers. A reduction of stigma against health care workers is thus warranted during the COVID-19 pandemic.

The use of unreliable forms of social media as information sources for COVID-19 can lead to the spread of misinformation and increased stigma against health care workers. This is especially true because various COVID-19–related rumors, stigma, and conspiracy theories have been circulating on the internet [9]. Most people rely on the internet for COVID-19 information [10,11]. Particularly, Twitter conversations pertaining to COVID-19 are characterized by the dissemination of stigmatizing messages [12,13]. Although the media, including newspapers, television, and websites, are important sources that can be used to promote health education and literacy, mass media and even health agencies have contributed to the spread of health misinformation that could circulate stigma on the internet [14]. In addition to the media, Twitter allows users to post short messages (tweets), “retweet” messages (reposts), send replies, and “like” messages by other users. Therefore, Twitter users are more likely to be exposed to misinformation and stigmatizing messages, which in turn may exacerbate fear and anxiety and result in the further circulation of such messages [9]. Adolescents and young adults have been using social networking services more frequently since the COVID-19 pandemic, as pandemic-related restrictions have substantially changed their social lives due to school closures [15]. A previous study indicated that the level of anxiety about COVID-19 differed according to the type of preferred news source [16]. However, no studies have examined the association between the types of COVID-19 information sources and stigma against health care workers. An understanding of this association will provide a basis for developing strategies aimed at reducing stigma.

This study, thus, investigated the association between COVID-19 information sources and stigma against health care workers among college students during the pandemic. We hypothesized that college students who used Twitter as a COVID-19 information source would show a more stigmatic attitude toward health care workers than those who do not use Twitter.

## Methods

### Study Design

This cross-sectional, observational study was conducted using Google Forms, which is a web-based tool that allows data collection through personalized surveys. An anonymous questionnaire was uploaded and shared through an invitation email to potential participants.

### Setting

On August 18, 2020, the survey link was shared with teachers from 8 colleges in the Tohoku region of Japan. The link provided a detailed explanation of the study purpose instructions. Subsequently, the teachers emailed the invitation link with the explanatory documents to the students. Participants aged ≥20 years were requested to complete the questionnaire on October 31, 2020.

The first page of the website contained details on the voluntary nature of the participation and protection of personal information. After reading the introduction, students indicated their consent to participate by clicking on the link to start the survey. The “Limit to 1 response” function of Google Forms was enabled to prevent respondents from completing the form more than once.

### Participants

We used purposive and convenience sampling to select colleges and departments with (1) nursing students who underwent on-the-job training in hospitals and (2) students from other departments. The other departments were selected to ensure gender ratios similar to those of students in the nursing departments.

Nursing and other students were recruited to compare the level of stigma against health care workers. We assumed that nursing students would show less stigmatic attitudes than other students, because they are expected to become professional nurses and, therefore, have more psychological proximity with other health care workers.

### Measurements

The questionnaire included questions regarding stigmatic attitudes against health care workers, COVID-19 information sources, perceived vulnerability to infection, department, age, sex, and contact with COVID-19 patients. The questions and response options of stigma against health care workers were developed for this study (Table S1 in Multimedia Appendix 1).

Stigma against health care workers was evaluated using a modified version of the Japanese version of the Social Distance Scale (SDSJ) [17]. The original Social Distance Scale was developed based on the Keyed Favorable Response and Ego-Involvement Ratings of Scale [18], which is used to assess the level of stigmatic attitude toward patients with schizophrenia. It contains 8 stigma-related items, which are rated on a 4-point Likert scale, and the scale has good reliability and validity [17]. For this study, we replaced “patients with schizophrenia” in each item with “health care workers and their families who are performing infectious disease management with an unestablished treatment regimen.” The total score on the 8 items ranged from 0 to 24; higher scores indicated more stigmatic attitudes.

The 8 types of COVID-19 information sources were listed in the questionnaire. Participants were asked to check any source, if applicable. The list was developed by a research panel to contain a range of social media platforms, including newspapers, television news streams, television tabloid talk shows, news websites, Twitter, the websites of public health agencies (eg, Ministry of Health, Labour and Welfare), Instagram, and Facebook.

Perceived vulnerability to infection was evaluated using the Perceived Vulnerability to Disease (PVD) scale [19]. The PVD contains 15 items, which are rated on a 7-point Likert scale from 1 to 7. It comprises 2 subscales: Perceived Infectability and Germ Aversion. Perceived Infectability refers to beliefs about immunological functioning and personal susceptibility to infectious diseases. Germ Aversion refers to aversive affective responses to situations that connote a relatively high likelihood of pathogen transmission. The Japanese version of the PVD is reported to have good reliability and validity [20]. In this study, the Cronbach α coefficient was .78 (95% CI .74-.82) for Perceived Infectability and .65 (95% CI .58-.71) for Germ Aversion. Although Perceived Infectability refers to one’s susceptibility to infection, Germ Aversion covers behaviors exerting emotional discomfort in a high-pathogen context, which in turn deters from the source of infection. Therefore, we assumed that stigma against health care workers would show a moderate positive correlation with Germ Aversion but not with Perceived Infectability.

Participants also answered items pertaining to their age, sex, department, and contact with COVID-19 patients. The presence of contact was assessed if (1) the respondent or their family members or friends had been infected with COVID-19; and if (2) the respondent or their family members or friends had close contact with infected persons. Based on the contact hypothesis [21,22], we assumed that individuals who directly interacted with COVID-19 patients would be less likely to hold stigmatic attitudes against health care workers. However, the number of individuals with direct contact was small (n=18). Therefore, we used the presence of contact for sensitivity analysis instead of including it in bivariate and multivariate analyses.

### Study Size

The required sample size was calculated using G*Power (version 3.1.9.7; Faul et al [23,24]). Based on a recent report on the use of Twitter among Japanese college students [25], we assumed the prevalence of Twitter as a COVID-19 information source to be 50% in this study. Assuming an α level of 5%, 95% power, and medium effect size (Cohen *d*=0.5) for a 2-tailed test, the minimum sample size was determined to be 210.

### Statistical Analysis

The validity and reliability of the modified SDSJ were examined. To test concurrent validity, the mean difference between nursing and other students was examined. The normality of distribution was assumed for total SDSJ score (Table S2 in Multimedia Appendix 1). The equality of variances between the 2 groups was also assumed (Table S3 in Multimedia Appendix 1). Therefore, 2-sided testing using 2-tailed students’ *t* test was used. To test convergent validity, Pearson correlation coefficient was calculated between the total SDSJ score and the subscales scores of the PVD. To test internal reliability, the Cronbach α coefficient and 95% CI [26] were calculated for the total SDSJ score.

Within the types of COVID-19 information sources, overlaps between Twitter and other major web-based platforms (news websites and the websites of public health agencies) were examined. To investigate the association between Twitter use and stigma against health care workers, the mean difference in SDSJ scores was examined by performing a 2-tailed *t* tests between participants who used Twitter as a COVID-19 information source and those who did not. Furthermore, a multiple Ordinary Least Square (OLS) regression analysis was performed using the SDSJ scores as the dependent variable and the types of information sources as independent variables. Participant characteristics, including age, sex, department, and PVD scores, were included as covariates. Diagnostic tests were conducted to test the assumptions of OLS regression, including the normality of the residuals, homoskedasticity, the absence of outliers, and low multicollinearity (Table S4 in Multimedia Appendix 1). Since some potential outliers were found, a sensitivity analysis of the multivariate model was performed by excluding them. Another sensitivity analysis was also conducted by excluding individuals with social contacts.

All analyses were conducted using Stata statistical software (version 17.0; StataCorp). The significance level was set at low (α=.1), medium (α=.05), and high (α=.01) [27]. As our primary endpoint was to assess the association between use of Twitter and SDSJ under multiple regression analysis adjusting for covariates, we did not apply *P* value adjustments for multiple hypothesis testing [28].

### Ethics Approval

The study protocol was approved by the Ethics Board of Tohoku University (2021-1-733) and was conducted in accordance with the Helsinki Declaration of 1975 (as revised in 2013).

## Results

### Participant Characteristics

A total of 281 participants completed the survey and were included in the final sample. The majority (86.7%, n=238) were women and the most frequent (50.9%, n=143) age was 21 years. There were 18 participants who reported coming into contact with COVID-19 (Table 1).

**Table 1:** Participant characteristics.

Variable, category		Participant (N=281), n (%)
**Sex**		
	Female	238 (86.7)
**Age (year)**		
	20	86 (30.6)
	21	143 (50.9)
	≥22	52 (18.5)
**Department**		
	Nursing	104 (37)
	Rehabilitation	70 (24.9)
	Psychology	63 (22.4)
	Other	44 (15.7)
**Contact with COVID-19**		
	Any of the experiences below	18 (6.4)
	Family members or friends had close contact with an infected person	13 (4.6)
	I had close contact with an infected person	1 (0.4)
	Family members or friends had been infected with COVID-19	5 (1.8)
	I had been infected with COVID-19	0 (0)

### Stigma Against Health Care Workers

The mean total SDSJ score in overall sample was 7.9 (SD 4.7). Nursing students had a significantly lower mean total score (mean 6.0, SD 4.5) than other students (mean 8.9, SD 4.5; *t*_218.48_=5.18, *P*<.001). The mean total SDSJ score did not differ between participants with social contact (mean 8.6, SD 1.0) and those without (mean 7.8, SD 0.3; *t*_20.16_=0.73, *P*=.47).

Pearson correlation coefficients were –0.03 (*P*=.62) between the SDSJ and PVD Perceived Vulnerability scores and 0.33 (*P*<.001) between the SDSJ and PVD Germ Aversion scores. The Cronbach α coefficient of the SDSJ score was high (α=.83; 95% CI .80-.86). In summary, the modified SDSJ demonstrated satisfactory concurrent, convergent, and internal validity.

### COVID-19 Information Sources

Half (49.5%, 139/281) of the participants used Twitter as a COVID-19 information source (Table 2). Since less than 10% of participants used newspapers (8.5%, n=24), Instagram (3.2%, n=9), or Facebook (0.4%, n=1), we excluded these sources from the following multivariate analysis.

**Figure 1:**
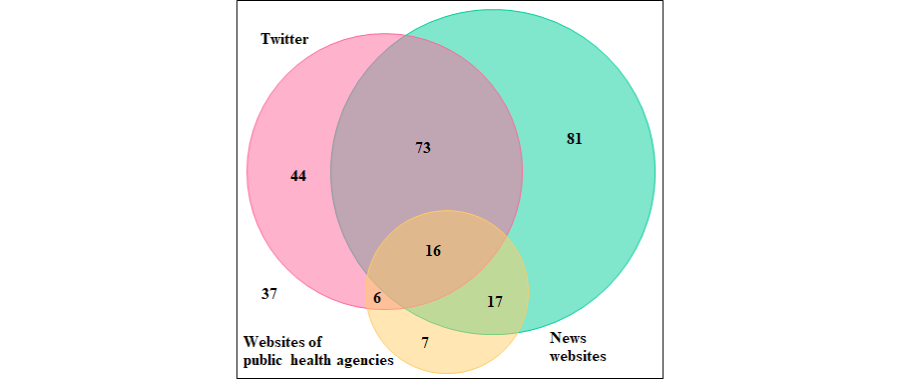
Venn diagram of overlaps between Twitter and other major web-based platforms (news websites and the websites of public health agencies).

In total, 89 participants reported using both Twitter and news websites, of which 16 participants also used the websites of public health agencies. Further, 44 participants used Twitter, but not news websites or the websites of public health agencies (Figure 1).

The total SDSJ score did not differ between Twitter users (mean 8.1, SD 4.4) and those who did not use Twitter (mean 7.6, SD 5.0; *t*_276.63_=0.97, *P*=.33).

**Table 2:** Prevalence of COVID-19 information sources.

Type, category		Participant (N=281), n (%)
Newspaper		24 (8.5)
**Television**		
	News stream	218 (77.6)
	Tabloid talk show	71 (25.3)
**Web-based source**		
	News website	187 (66.5)
	Twitter	139 (49.5)
	Website of public health agencies (eg, Ministry of Health, Labour and Welfare)	46 (16.4)
	Instagram	9 (3.2)
	Facebook	1 (0.4)
	Other social networking services	13 (4.6)

### Association Between Stigma and Information Sources

A multiple regression analysis showed that the total SDSJ scores were lower among participants using the websites of public health agencies (*P*=.008), nursing students (*P*<.001), and those with lower Germ Aversion scores (*P*<.001; Table 3). The use of Twitter was not associated with SDSJ scores (*P*=.58).

The results of OLS diagnostic tests showed that the following assumptions were met: the normality of the residuals, homoskedasticity, and low multicollinearity (Table S4 in Multimedia Appendix 1). However, there were 2 potential outliers (Table S4 in Multimedia Appendix 1). A sensitivity analysis that excluded the 2 individuals did not change the results (Table S5 in Multimedia Appendix 1).

Another sensitivity analysis that excluded individuals (n=18) who came into close contact with a patient with COVID-19 did not alter the results (Table S5 in Multimedia Appendix 1).

**Table 3:** Multiple linear regression analysis of stigma against health care workers^a,b^.

Variable, category		Coefficient (95% CI)	*P* value
**COVID-19 information source**			
	Television news stream	0.86 (–0.40 to 2.12)	.18
	Television tabloid show	0.51 (–0.70 to 1.72)	.41
	News website	0.60 (–0.45 to 1.66)	.26
	Twitter	0.29 (–0.74 to 1.31)	.58
	Websites of public health agencies	–1.84 (–3.20 to –0.49)	.008
**Sex**			
	Male	1.07 (–0.38 to 2.52)	.15
**Age (year)**			
	20	0.33 (–1.21 to 1.88)	.67
	21	–0.25 (–1.64 to 1.14)	.72
	≥22	reference	reference
**Department**			
	Nursing	–3.04 (–4.11 to –1.98)	<.001
**Perceived vulnerability to infection**			
	Perceived Infectability	–0.13 (–0.63 to 0.37)	.61
	Germ Aversion	1.85 (1.31-2.39)	<.001

^a^Stigma against health care workers was evaluated using the modified Japanese language version of the Social Distance Scale; the total score ranges from 0 to 24.

^b^Perceived vulnerability to infection was evaluated using the Japanese version of the Perceived Vulnerability to Disease scale; total scores range from 1 to 7 for both Perceived Infectability and Germ Aversion.

## Discussion

With increasing concerns about the stigma against health care workers during the COVID-19 pandemic, strategies to reduce stigma are the need of the hour, considering the prevalence of Twitter and other information sources for information related to COVID-19. Thus, this study examined the association between the types of information source and stigma against health care workers among college students.

### Principal Findings

Contrary to our hypothesis, Twitter use was not associated with the stigma against health care workers. The survey was conducted between August and October 2020 when the daily number of new COVID-19 cases in Japan ranged from 219 to 1178. This period is a few months after the onset of the outbreak, and our participants may have had a lower level of fear than that from February to April 2020 [9,12,13]. In the acute phase of the psychological response to a crisis, such as the Great East Japan Earthquake on March 11, 2011, Twitter messages diffused rumors and misinformation [29]. However, the level of anxiety expressed in Twitter messages appears to return to normal over time [30]. This shift to Twitter for content has also been observed for COVID-19 [31]. In addition, several students aged ≥20 years in the Tohoku region might have experienced the March 11 earthquake when they were children, resulting in their learned mindset to treat Twitter messages with caution.

College students who used the websites of public health agencies as information sources reported significantly less stigmatic attitudes than those who did not (*P*=.008) in the multiple regression analysis. Fact-checking and directing users to credible information sources from the websites of public health agencies can prevent the further spread of misinformation [32]. News websites also disseminate official announcements from public health agencies. In this study, substantial overlaps were observed between the users of the websites of public health agencies, news websites, and Twitter. This finding is consistent with a previous study [10]. Furthermore, public health agencies also have Twitter accounts, and each message is limited to 140 Japanese characters. Their messages usually include a URL to official website pages that contain longer texts. The accuracy of the information held by Twitter users may vary between those who only read the message and those who click on the link to access the website. Adequate communication strategies should be embedded in reliable information from trusted sources [31,33]. Considering that Twitter was a popular COVID-19 information source, access to information curated by public agencies may help reduce exposure to misinformation and stigma against health care workers. In addition to accurate information, “hero” messaging [34] should be reserved for health care workers in public policy. Health care workers were deemed essential frontline heroes during the COVID-19 crisis. Such perceptions can mitigate stigmatic attitudes toward health care workers. Furthermore, positive video messages embedded in tweets may enable young people to engage in parasocial interactions with health care workers, which in turn may help change their negative beliefs about health care workers based on the parasocial contact hypothesis [22,35].

Stigma against health care workers, as measured by the modified SDSJ, was significantly lower among nursing students (*P*<.001) and students with lower germ aversion (*P*<.001). Unlike Germ Aversion, we found very little statistical evidence that Perceived Infectability was associated with stigma against health care workers using the total SDSJ score. These associations were consistent with our assumption that the modified scale is valid.

### Strengths and Limitations

The strength of this study lies in the examination of stigma against health care workers in the context of COVID-19 and social media, thus addressing a gap in the literature. A limitation was the lack of information accuracy and limited data on the intensity of social media use among participants. In addition, the cross-sectional design precludes causal inferences between stigma and the types of information sources. The use of websites of public health agencies might indicate the high health literacy and low stigmatic attitudes of students.

### Conclusions

A few months after the onset of the COVID-19 pandemic, nearly half of the college student population used Twitter as an information source. Our findings showed that the level of stigma against health care workers did not differ according to Twitter use. Students who used the websites of public health agencies reported less stigmatic attitudes than those who did not. These results imply that directing people to credible COVID-19 information sources from public agencies may prevent the formation of stigmatic attitudes against health care workers. An effective strategy for the induction of access to credible information sources should be explored for integration into Twitter and other widespread web-based platforms.

## Appendix

Multimedia Appendix 1 Supplementary tables on (1) questions and response options used in the online survey; (2) test of the normality of scores; (3) Ordinary Least Square regression diagnostic tests; (4) sensitivity analysis: multiple linear regression analysis of stigma against health care workers, excluding possible outliers (N=279); and (5) sensitivity analysis: multiple linear regression analysis of stigma against health care workers, excluding individuals who came in close contact with a patient with COVID-19 (N=263).

## Abbreviations

OLS: Ordinary Least Square
PVD: Perceived Vulnerability to Disease
SDSJ: Japanese version of the Social Distance Scale
