# Supplementary material for: The Association Between COVID-19 Information Sources and Stigma Against Health Care Workers Among College Students: Cross-sectional, Observational Study
Source: JMIR Form Res. 2022 Jul 7;6(7):e35806. doi: 10.2196/35806 (PMC9273044; doi:10.2196/35806)
Supplement: Multimedia Appendix 1 [file formative_v6i7e35806_app1.docx]

**Multimedia Appendix**

**Table S1. Questions and response options for used in the online survey**

| Stigma against healthcare workers | Response option |
| --- | --- |
| 治療法が見つかっていない感染症を扱う医療者と家族とは付き合わないのが一番である  It is best not to associate with a person | 4 そう思う Agree  3 ある程度そう思う Agree to some extent  2 あまり思わない Disagree to some extent  1 そう思わない Disagree |
| 治療法が見つかっていない感染症を扱う医療従事者と家族を避けるのは間違いである  It is wrong to shy away from a person | 4 そう思う Agree  3 ある程度そう思う Agree to some extent  2 あまり思わない Disagree to some extent  1 そう思わない Disagree |
| 治療法が見つかっていない感染症を扱う医療従事者と家族の近所で暮らすことになったら、それは私にとって苦になるだろう  It would bother me to live near a person | 4 そう思う Agree  3 ある程度そう思う Agree to some extent  2 あまり思わない Disagree to some extent  1 そう思わない Disagree |
| 私は、治療法が見つかっていない感染症を扱う医療従事者の家族が運転するタクシーには乗りたくない  I would not ride in a taxi driven by someone | 4 そう思う Agree  3 ある程度そう思う Agree to some extent  2 あまり思わない Disagree to some extent  1 そう思わない Disagree |
| 私は、治療法が見つかっていない感染症を扱う医療従事者の家族は雇いたくない  I would rather not hire a person | 4 そう思う Agree  3 ある程度そう思う Agree to some extent  2 あまり思わない Disagree to some extent  1 そう思わない Disagree |
| 治療法が見つかっていない感染症を扱う医療従事者の家族は学校で教えることを許可されるべきではない  A person who … should not be allowed to teach at school | 4 そう思う Agree  3 ある程度そう思う Agree to some extent  2 あまり思わない Disagree to some extent  1 そう思わない Disagree |
| 私は、ベビーシッターを雇うとき、治療法が見つかっていない感染症を扱う医療従事者の家族であってもかまわない  If I needed a baby sitter, I would be willing to hire a woman | 4 そう思う Agree  3 ある程度そう思う Agree to some extent  2 あまり思わない Disagree to some extent  1 そう思わない Disagree |
| もし、治療法が見つかっていない感染症を扱う医療者やその家族と自分の娘が結婚したいと言ったならば、娘がどうであれ私は結婚に反対するだろう  I would be against any daughter of mine marrying a man | 4 そう思う Agree  3 ある程度そう思う Agree to some extent  2 あまり思わない Disagree to some extent  1 そう思わない Disagree |

**Table S2. Test of the normality of SDSJ scores**^a^

|  | Skewness | P-value | Kurtosis | P-value | Adjusted χ^2^ (2) | P-value |
| --- | --- | --- | --- | --- | --- | --- |
| SDSJ | 0.22 | .126 | 2.73 | .377 | 3.14 | .208 |

^a^Stigma against healthcare workers was evaluated using the modified Japanese language version of the Social Distance Scale (SDSJ); the total score ranges from 0 to 24.

**Table S3. Test of the equality of variance in SDSJ scores between two groups**^a^

|  | Yes |  | No |  |  |  |
| --- | --- | --- | --- | --- | --- | --- |
|  | N | Mean (SD) | N | Mean (SD) | Test statistic | P-value |
| Nursing | 104 | 6.0 (4.5) | 177 | 8.9 (4.5) | F (176, 103) = 1.03 | .883 |
| Social contact | 18 | 8.6 (4.1) | 263 | 7.8 (4.7) | F (17, 262) = 0.77 | .534 |
| Use of Twitter | 139 | 8.1 (4.4) | 142 | 7.6 (5.0) | F (141, 138) = 1.26 | .178 |

^a^Stigma against healthcare workers was evaluated using the modified Japanese language version of the Social Distance Scale (SDSJ); the total score ranges from 0 to 24.

**Table S4. Ordinary Least Square (OLS) regression diagnostic tests**

| Assumption | Test | Test statistic |
| --- | --- | --- |
| Normality of residuals | Skewness and kurtosis test | Adjusted χ^2^ (2) = 4.10, P = .129 |
| Homoskedasticity | Breusch–Pagan/Cook–Weisberg test | χ^2^ (1) = 1.88, P = .170 |
| Absence of outliers | Absolute value of studentized residual < 3.0 | Two cases exceeded (3.15 and 3.40) |
| Multicollinearity | Variance Inflation Factor (VIF) of covariate < 5 |  |
|  | TV news stream | VIF = 1.17 |
|  | TV tabloid show | VIF = 1.17 |
|  | News website | VIF = 1.05 |
|  | Twitter | VIF = 1.11 |
|  | Websites of public health agencies | VIF = 1.07 |
|  | Sex, male | VIF = 1.16 |
|  | Age, years, 20 | VIF = 2.16 |
|  | Age, years, 21 | VIF = 2.05 |
|  | Department, nursing | VIF = 1.12 |
|  | Perceived infectability | VIF = 1.06 |
|  | Germ Aversion | VIF = 1.06 |

**Table S5. Sensitivity analysis: Multiple linear regression analysis of stigma against healthcare workers, excluding possible outliers (N = 279)** ^a,b^

| Variable | Category | Coefficient (95%CI) | P-value |
| --- | --- | --- | --- |
| COVID-19 information source | TV news stream | 0.76 (-0.46, 1.97) | .221 |
|  | TV tabloid show | 0.60 (-0.57, 1.76) | .315 |
|  | News website | 0.89 (-0.14, 1.91) | .089 |
|  | Twitter | 0.48 (-0.51, 1.46) | .344 |
|  | Websites of public health agencies | -1.73 (-3.03, -0.42) | .010 |
| Sex | Male | 0.79 (-0.62, 2.20) | .269 |
| Age, years, reference = 22 years or older | 20 | 0.33 (-1.16, 1.83) | .661 |
|  | 21 | -0.43 (-1.77, 0.92) | .532 |
| Department | Nursing | -3.18 (-4.21, -2.15) | <.001 |
| Perceived vulnerability to infection | Perceived Infectability | 1.80 (1.28, 2.32) | <.001 |
|  | Germ Aversion | 0.90 (-2.50, 4.29) | .603 |

^a^Stigma against healthcare workers was evaluated using the modified Japanese language version of the Social Distance Scale (SDSJ); the total score ranges from 0 to 24.

^b^Perceived vulnerability to infection was evaluated using the Japanese version of the Perceived Vulnerability to Disease scale; total scores range from 1 to 7 for both Perceived Infectability and Germ Aversion.

**Table S6. Sensitivity analysis: Multiple linear regression analysis of stigma against healthcare workers, excluding individuals who came in close contact with a patient with COVID-19 (N = 263)** ^a,b^

| Variable | Category | Coefficient (95%CI) | P-value |
| --- | --- | --- | --- |
| COVID-19 information source | TV news stream | 0.76 (-0.58, 2.11) | .264 |
|  | TV tabloid show | 0.70 (-0.57, 1.97) | .278 |
|  | News website | 0.56 (-0.56, 1.68) | .324 |
|  | Twitter | 0.09 (-0.99, 1.18) | .868 |
|  | Websites of public health agencies | -1.77 (-3.21, -0.33) | .016 |
| Sex | Male | 1.11 (-0.44, 2.66) | .159 |
| Age, years, reference = 22 years or older | 20 | 0.28 (-1.37, 1.93) | .742 |
|  | 21 | -0.39 (-1.86, 1.09) | .605 |
| Department | Nursing | -2.93 (-4.04, -1.83) | <.001 |
| Perceived vulnerability to infection | Perceived Infectability | 1.92 (1.34, 2.50) | <.001 |
|  | Germ Aversion | 0.37 (-3.34, 4.08) | .843 |

^a^Stigma against healthcare workers was evaluated using the modified Japanese language version of the Social Distance Scale (SDSJ); the total score ranges from 0 to 24.

^b^Perceived vulnerability to infection was evaluated using the Japanese version of the Perceived Vulnerability to Disease scale; total scores range from 1 to 7 for both Perceived Infectability and Germ Aversion.
